# Endothelial Progenitor Cells in Life, Pregnancy and Disease

**DOI:** 10.1017/erm.2025.10015

**Published:** 2025-08-22

**Authors:** Bianca Schröder-Heurich, Julia Beckmann, Frauke von Versen-Höynck

**Affiliations:** 1 Department of Gynaecology and Obstetrics, Gynaecology Research Unit, Hannover Medical School, Hannover, Germany; 2 Department of Gynaecology and Obstetrics, Division of Reproductive Medicine and Gynaecologic Endocrinology, Hannover Medical School, Hannover, Germany

**Keywords:** ageing, cardiovascular risk, ECFCs, endothelial progenitor cells, EPCs, maternal health, neonatal health, pregnancy

## Abstract

Endothelial progenitor cells (EPCs) are key regulators of vascular homeostasis in both health and disease, playing a crucial role in regenerating the human vascular lining throughout life. These circulating cells can differentiate into mature endothelial cells and are increasingly recognized as important biological markers of vascular function and cumulative risk for various diseases, including cardiovascular conditions. In recent decades, the role of EPCs, particularly the endothelial colony-forming cells (ECFCs) subtype, in pregnancy-related disorders and maternal and neonatal endothelial health has garnered significant attention. Evidence suggests that ECFCs may serve as predictor of future endothelial health in women and their offspring following pregnancy complications, making them particular relevant for research and therapeutic applications in adulthood, as well as potential indicators of vascular health. This review summarizes the evidence on EPCs, specifically ECFCs, as biomarkers of endothelial health in pregnancy, pregnancy-related diseases and ageing, with a focus on maternal and foetal endothelial abnormalities that may serve as prognostic factors for the development of future diseases.

## Introduction

Angiogenesis and vasculogenesis are processes through which EPCs, a diverse group of endothelial precursor cells, contribute to vascular repair and regeneration. Overlapping haematopoietic and endothelial lineage characteristics have defined EPCs since their discovery in the late 1990s, but their precise identity remains debated. Researchers identified special EPC subtypes based on origin, markers and function (Refs 1, 2). First described as capable of differentiating into mature endothelial cells *in vitro*, EPCs play a critical role in neovascularization in ischaemic tissues (Ref. 1). Subsequent studies have elucidated the multifaceted roles of EPCs, highlighting their significance in maintaining vascular integrity and their potential as predictive biomarkers for various pathophysiological conditions. EPCs, especially the subtype of ECFCs, have been linked to vascular homeostasis and are increasingly recognized for their involvement in pregnancy-related disorders. This underscores their relevance in maternal and neonatal health and offers insights into their prognostic value for long-term cardiovascular (CV) outcomes (Refs 3–5).

### Putative endothelial progenitor cell types

Bone-marrow-derived EPCs are described in the literature as circulating within the bloodstream and can be derived from human peripheral blood mononuclear cells and the umbilical cord blood (UCB) of newborns (Refs 6–8)^1^.

**Figure 1.**
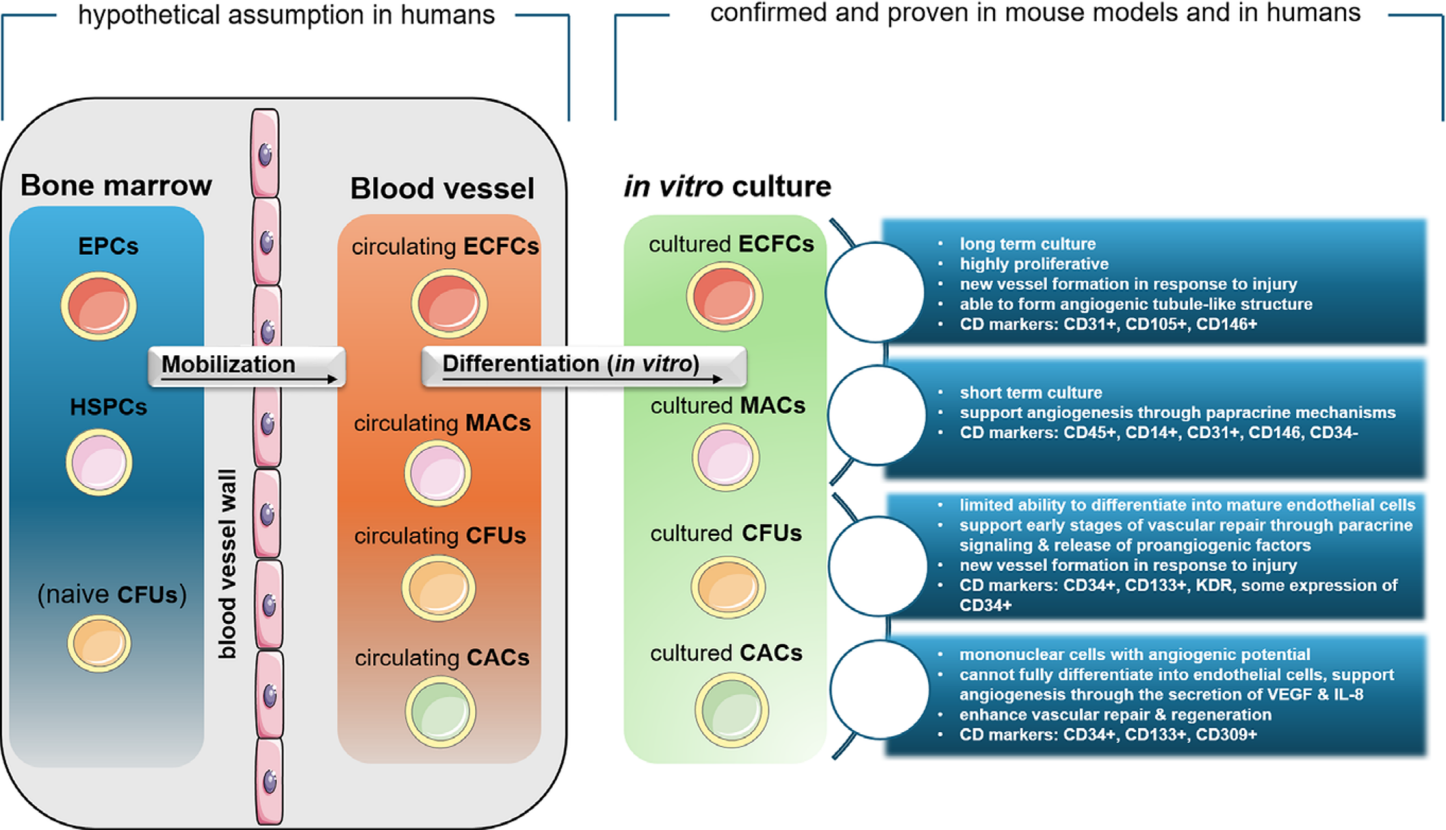
Subtypes of endothelial progenitor cells (EPCs), including ECFCs, CFUs, CACs and MACs, illustrating their origin, CD markers and functional roles in angiogenesis and vascular repair. ECFCs are primarily involved in vessel formation, while CFUs and CACs contribute to vascular regeneration and macrophage function. MACs are linked to inflammation and angiogenesis. ECFCs: endothelial colony forming cells; CFUs: colony-forming units-Hill, CACs: circulating angiogenic cells, MACs: myeloid angiogenic cells; EPCs: endothelial progenitor cells; HSPCs: haematopoietic stem and progenitor cells.

Beyond these sources, EPCs have been localized in various tissues, including vessel walls, umbilical cord, adipose tissue, cardiac tissue, placenta, spleen and neural tissue, underscoring their widespread distribution and potential functional diversity (Refs 9–11). Further, they play a crucial role in postnatal vasculogenesis, wound healing, recovery from myocardial infarction and the treatment of limb ischaemia (Ref. 12). Various methods are employed to characterize EPCs, including phagocytosis of acetylated low-density lipoprotein, the binding of lectin and the expression of surface markers (clusters of differentiation, CD) CD34, CD133 and the endothelial marker KDR (kinase insert domain receptor). During further differentiation, EPCs lose CD133 expression and upregulate endothelial markers such as CD31, vascular endothelial cadherin (VE-cadherin) and von Willebrand factor (vWF) (Refs 1, 7, 13). According to current consensus, EPCs are classified into two main groups: (1) cells directly detected in peripheral blood using flow cytometry, and (2) EPCs derived from *in vitro* culture of mononuclear cells. Culture-derived EPCs include ECFCs, which possess proliferative and vessel-forming capacity, and myeloid angiogenic cells (MACs), which promote vascular growth via paracrine mechanisms (Ref. 14). ECFCs are true endothelial progenitors with clonal proliferative capacity and the ability to form capillary-like structures *in vitro* and functional blood vessels *in vivo* (Refs 6, 15) (Figure 1). They express markers such as CD34, CD133, vascular endothelial growth factor receptor (VEGFR) 2 (also known as KDR), CD31, VE-cadherin and vWF, while being negative for haematopoietic markers like CD45 and CD14 (Ref. 16).

MACs, formerly referred to as early EPCs, circulating angiogenic cells (CACs), or colony-forming unit endothelial cells (CFU-ECs), are haematopoietic in origin and do not differentiate into endothelial cells. Instead, they promote angiogenesis indirectly through the secretion of cytokines such as vascular endothelial growth factor (VEGF) and interleukin (IL)-8. MACs express CD45, CD14 and CD31, but lack specific endothelial markers like CD146 or VE-cadherin. Although they are not true EPCs, they contribute to vascular regeneration via paracrine mechanisms (Refs 14, 15, 17, 18).

Together, ECFCs and MACs represent distinct cell populations with different lineages and mechanisms of promoting angiogenesis and vascular repair (Ref. 19). Figure 1 provides an overview of EPC subtypes, highlighting their function, origins and associated CD markers. To reflect the current state of evidence regarding their existence, the figure is subdivided into two categories: (1) hypothetical assumptions in humans, and (2) confirmed and proven in mouse models and humans.

### Endothelial-colony-forming cells

ECFCs play a pivotal role in maintaining vascular homeostasis and actively contribute to vascular repair in response to injury. Under physiological conditions, they support endothelial integrity and renewal, while in pathological circumstances, such as ischaemia or tissue damage, their robust proliferative capacity and responsiveness to pro-angiogenic signals facilitate neovascularization and endothelial restoration (Refs 20, 21).

Unlike early outgrowth EPCs, which contribute indirectly to angiogenesis through paracrine signalling, ECFCs exhibit distinct endothelial characteristics, including a strong proliferative capacity, the ability to form vessel-like structures *in vitro* and direct integration into vasculature *in vivo.* ECFCs are derived from the vascular endothelial lineage rather than haematopoietic progenitors, as indicated by their distinct cell surface markers, including CD31, CD34 and CD144, and the absence of haematopoietic markers such as CD45. Additionally, ECFCs are clonogenic and capable of producing endothelial colonies, distinguishing them from other EPC subtypes. Their ability to respond to pro-angiogenic signals further underscores their critical role in processes such as wound healing, tissue repair and adaptation to ischaemia (Ref. 22).

Conversely, ECFCs isolated from human neonatal sources, such as UCB, demonstrate greater proliferative and angiogenic potential than compared to adult peripheral blood, highlighting their therapeutic promise. UCB-derived ECFCs are being explored in regenerative medicine for their ability to restore vascular networks in ischaemic tissues and enhance the efficacy of engineered grafts (Ref. 23).

Research on ECFCs continues to progress, with ongoing efforts to harness their regenerative potential in clinical applications. However, challenges remain in standardizing isolation methods and optimizing their expansion for therapeutic use. A detailed overview of the various EPC isolation techniques is provided in the review by Chen *et al*. (Ref. 24).

To pave the way for clinical implementations, it is essential to decipher how ECFCs exert their regenerative effects at the cellular and molecular level. These cells promote angiogenesis and vascular repair by interacting with various cell types and molecular signals. They differentiate into endothelial cells and interact with other endothelial cells, vascular smooth muscle cells and pericytes to form new blood vessels. Moreover, ECFCs engage with macrophages and haematopoietic stem cells to facilitate vascular repair. ECFCs are activated by growth factors such as VEGF, fibroblast growth factor (FGF) and platelet-derived growth factor (PDGF), which regulate their migration and differentiation (Ref. 12). VEGF plays a crucial role in EPC mobilization by promoting their release from the bone marrow and facilitating their integration into damaged vascular areas (Ref. 1). The expression of adhesion molecules like ICAM-1 and VCAM-1 on damaged endothelial cells promotes the attachment and incorporation of ECFCs into the injured endothelium (Ref. 25). Additionally, ECFCs play a role in responding to hypoxic conditions and the extracellular matrix, which supports their function and stability. These diverse interactions are crucial for the formation of stable and functional blood vessels (Refs 26, 27). Hypoxic conditions at the injury site further enhance ECFC recruitment through the activation of hypoxia-inducible factor (HIF)-1α, which upregulates pro-angiogenic factors, driving the repair process (Refs 20, 28).

However, external factors like lifestyle, comorbidities and environmental influences significantly affect their efficacy. For instance, regular exercise boosts EPC mobilization and functionality, whereas smoking or hyperlipidaemia reduce their regenerative potential through oxidative stress and inflammation (Refs 29, 30). A notable example of a potential therapeutic agent that promotes EPC production is relaxin, a peptide hormone that plays a crucial role during pregnancy but is also involved in the menstrual cycle. Relaxin stimulates the mobilization of EPCs from the bone marrow by increasing vascular permeability and improving endothelial function, which may facilitate vascular repair and angiogenesis. Segal *et al.* demonstrated that relaxin enhances the release of EPCs from the bone marrow, potentially aiding in the healing of vascular damage (Ref. 31).

### EPCs during pregnancy

Research has shown significant variability in EPCs behaviour during pregnancy, highlighting their dynamic regulation by gestational age, hormonal influences and maternal–foetal interactions. Research by Sugawara *et al*. established that circulating EPC levels positively correlate with oestrogen concentrations. Oestrogen facilitates EPC mobilization from the bone marrow and delays their senescence through upregulation of VEGF production. This suggests a dual role for oestrogen in sustaining EPCs functionality and promoting vascular repair mechanisms (Ref. 32).

Subsequent investigations using flow cytometry confirmed an increase in EPC numbers early in pregnancy, peaking during the third trimester. Throughout gestation, circulating EPCs, characterized by markers such as CD34, CD133 and VEGFR2, exhibit dynamic changes in their expression profiles. In the first trimester, there is a notable rise in CD34(+)CD133(+)VEGFR2(+) cells, indicating heightened vasculogenic potential, likely in response to the increased demands of placental and foetal development. Consistent with this, Singh *et al.* reported that circulating EPC levels, defined as CD45(d)CD34(+)KDR(+)CD133 cells, are significantly higher during healthy pregnancy compared to the non-pregnant control group (699 versus 398 cells/ml) (p = 0.03) (Ref. 33).

As gestation progresses into the second trimester, there is a marked and continuous increase in both the absolute number and functional capacity of EPCs, contributing to vascular remodelling and placental angiogenesis. By the third trimester, the peak in EPCs (defined as CD34(+)CD133(+)VEGFR2(+)) concentration reflects the culmination of vascular adaptation processes necessary for sustaining foetal growth and preparing for parturition. The temporal changes in EPC marker expression suggest a tightly regulated mechanism of vascular homeostasis, underscoring the critical role of EPCs in maternal vascular health throughout pregnancy (Refs 3, 34). However, contrasting findings revealed an elevation in EPCs during early pregnancy, followed by a decline in mid-to-late pregnancy, aligning with angiogenic activity peaks (Ref. 35). Discrepancies in the temporal regulation of EPCs, such as those reported by Attar *et al*., who observed fluctuating CFU levels throughout pregnancy, underscore the complexity of EPC dynamics. In their study, they described EPCs as putative progenitor cells derived from human blood, emphasizing their crucial role in vascular homeostasis. Additionally, most studies indicate an increase in CACs in the maternal circulation as gestation progresses in normal pregnancies (Refs 12, 33, 36).

Similarly, Parsanezhad and co-workers found no significant differences in CFU-EC and ECFC levels between pregnant and non-pregnant individuals, further highlighting the need for refined methodologies to resolve these inconsistencies (Refs 36, 37). UCB-derived ECFCs also displayed variability based on gestational age (Ref. 38).

A recent study introduced an innovative method for isolating human endothelial cells from first-trimester umbilical cords (First-Trimester Umbilical Endothelial Cells, FTUECs). The ‘flow-through’ isolation technique offers a gentle approach that enables the acquisition of highly purified endothelial cells. This method is of particular interest as the isolated cells exhibit a CD34-positive, juvenile endothelial phenotype, reflecting the early stages of placental vascular development. FTUECs are characterized by their ability to expand and be passaged, making them a valuable model for studying developmental processes and the effects of adverse conditions during early pregnancy (Ref. 39).

Interestingly, maternal peripheral blood EPC populations appear to include foetal progenitor cells, as evidenced by the presence of the foetal *SRY* gene in specific EPC subsets. Placenta-derived ECFCs contribute further complexity, as macrovascular cells are foetal-derived, while microvascular cells originate from the mother (Refs 40, 41). Although placental and UCB-derived ECFCs exhibit similar angiogenic capacities and gene expression profiles, it remains unclear if placental ECFCs represent the primary source of EPCs within the maternal or foetal circulations (Refs 34, 37).

### EPC throughout the lifespan

Throughout the human lifespan, EPCs are integral to vascular maintenance and repair, although their abundance and functionality vary across different stages of life. During foetal development, EPCs are highly proliferative and exhibit enhanced angiogenic potential, playing a critical role in vascular formation and remodelling. This functional peak supports the rapid vascular expansion required in the developing organism.

In adults, the capacity and functionality of the EPC subtype of ECFCs decline with age, largely due to decreased sensitivity to VEGF (Ref. 42). This reduction is associated with impaired vascular repair and increased susceptibility to cardiovascular diseases (CVD).

In neonates and young adults, EPCs exhibit high proliferative and angiogenic potential, facilitating efficient endothelial regeneration (Ref. 1). However, ageing is associated with a marked reduction in circulating EPCs, impaired migratory capacity and increased susceptibility to oxidative stress and inflammation. These changes are thought to contribute to endothelial dysfunction and the pathogenesis of age-related vascular diseases such as atherosclerosis and hypertension (Refs 2, 43). Given their role in vascular homeostasis, EPCs are being explored as both biomarkers of vascular ageing and targets for regenerative therapies (Ref. 2).

Reduced levels and functional impairment of EPCs have been shown in CAD patients, which may contribute to impaired vascularization in these patients (Ref. 44). The bone-marrow-derived cells, including EPCs demonstrated a progressive dysfunction associated primarily with cellular senescence, apoptosis and free radical accumulations. The bone marrow represents one of the most critical organs negatively impacted by ageing due to its role in serving as a haematopoietic stem cell/EPCs reservoir and providing cellular components of the immune system across the lifespan (Ref. 44). Conversely, interventions like physical exercise, statin therapy and administration of pro-angiogenic cytokines have been shown to partially restore EPC function and improve vascular outcomes in aged subjects (Refs 2, 45).

## The role of EPCs in disease, pregnancy-related complications and ageing

### EPCs in disease pathogenesis

Over the past decades, EPCs have been described as playing a role in various diseases (Ref. 46). In ischaemic conditions, such as myocardial infarction and peripheral artery disease, ECFCs contribute to restoring blood flow to damaged tissue by promoting angiogenesis (Ref. 16). In hypoxia and pulmonary arterial hypertension, ECFCs play a crucial role in the adaptive response to low oxygen levels and the development of new blood vessels in the lungs (Ref. 16). A role for ECFCs has been additionally described in cancer by providing the necessary blood supply for tumour growth and metastasis (Ref. 16). Further attributions have been made to diabetes mellitus, where ECFCs are affected by the hyperglycaemic environment, leading to impaired angiogenic capacity (Ref. 16). In patients with coronary artery disease (CAD), a reduced number of EPCs was associated with a higher incidence of CV events (Refs 47, 48).

**Figure 2.**
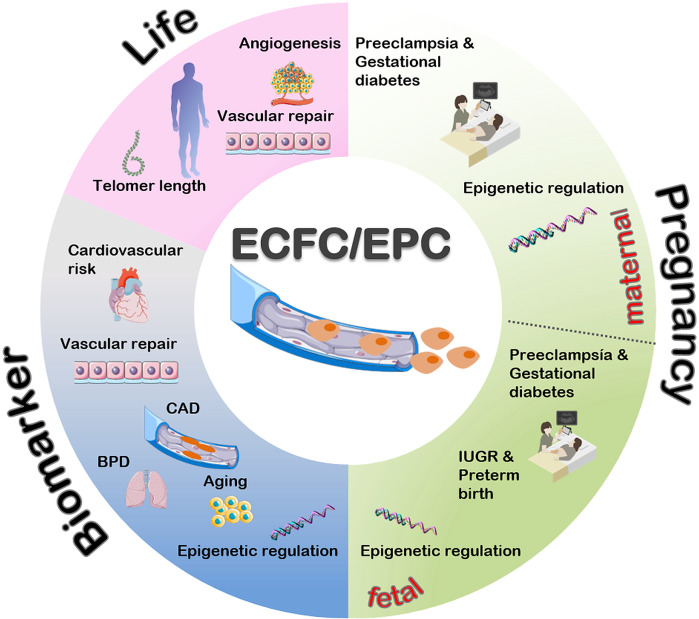
Functional role of EPCs/ECFCs as biomarker, during pregnancy and life. BPD: bronchopulmonary dysplasia; CAD: coronary artery disease; IUGR: intrauterine growth restriction.

Based on these findings, EPCs represent a promising group of cells that could serve as potential biomarkers, particularly in the CV field, due to their unique properties and function (Ref. 46). In the following sections, we will focus specifically on EPCs and ECFCs as a potential marker in pregnancy-related conditions and their impact on both mother and infant. Figure 2 provides an overview of the significance of EPCs and ECFCs in life, during pregnancy and as biomarkers.

### EPCs as a potential biomarker in pregnancy-related conditions – insights from the maternal side

The identification of biomarkers plays a crucial role in the early detection and risk stratification of diseases. However, there are few studies describing the role of EPCs in pregnancy-related conditions that might explain their contribution to future CV events or other pathologies. Table 1 provides an overview of the functionality and characterization of EPCs and ECFCs in pregnancy-related diseases. For the presentation and classification of EPCs, this review deliberately refrained from introducing a separate subclassification. Instead, where available in the original publication, the marker combinations and cell type designations used there were adopted.

**Table 1.** EPC/ECFC characteristics and main findings in pregnancy-related diseases and outcomes

Reference	Biological sample	Population	Cell surface markers	Mode of change
*Preeclampsia*				
Luppi et al.(Ref. 55)	PB	EPCs	CD34(+),CD309(+) and CD133(+),CD309(+)	↓ EPC number
Matsubara et al. (Ref. 26)	PB	EPCs (CACs)	CD34(+),CD133(+),CD309(+)	= EPC number ↑ EPC proliferation
Parsanezhad et al. (Ref. 28)	PB	CACs ECFCs	CD34(+),CD133(+),CD309(+),CD45(+) CD309(+,)CD45(−)	↑ CAC number ↑ ECFC number
Muñoz-Hernandez et al. (Ref. 81)	CB	ECFCs	CD31(+), CD45(−),CD90(−)	↓ ECFC colony number
Monga et al. (Ref. 83)	CB	EPCs	CD34(+),Cd133(+),Cd45(d),CD309(+)	= EPC number
*Gestational diabetes*				
Penno et al.(Ref. 57)	PB	EPCs	CD34(+)KDR(+)CD133(+)	↓ EPC number
Mordwinkin et al. (Ref. 58)	PB CB	EPCs EPCs	CD34(+),CD309(+),CD133(+) CD34(+),CD309(+),CD133(+)	↓ EPC number = EPC number
Ingram et al. (Ref. 63)	CB	ECFCs	n.A.	↓ ECFC colony number
Acosta et al. (Ref. 65)	PB CB PB/CB	CPCs CPCs CECs	CD34(+),CD133(+),CD45(+),CD31(+) CD34(+),CD133(+),CD45(+),CD31(+) CD34(+),CD133(−),CD45(−), CD31(+)	↓ CPC number at 24–32 weeks’ gestation ↓ CPC number = CEC number
*Preterm birth & bronchopulmonary dysplasia*				
Baker et al. (Ref. 82)	CB	CPCs ECFCs	CD34(+),CD45(d),CD31(+),CD133(+),CD14(−),CD235a(−) number of ECFC colonies per 10^7^ mononuclear cells	↓ CPC ECFC colony number ↓ ECFC colony number
Safranow et al. (Ref. 90)	PB/CB	early EPCs late EPCs	CD34(+), CD133(+),CD144(+) CD34(+), CD133(−),CD144(+)	↑ early and late EPC number
Machalinska et al. (Ref. 89)	PB	early EPCs	CD34(+),CD133(+),CD144(+)	↑ EPC number
Monga et al. (Ref. 83)	CB	EPCs	CD34(+),CD133(+),CD45(d),CD309(+)	= EPC number
Baker et al. (Ref. 91)	CB	EPCs	CD34(+),CD133(−),CD45(−), CD31(+),CD105(+), CD146(+),CD309(+)	= EPC number ↑ ECFC colony number
Bui et al. (Ref. 92)	PB	EPCs	CD34(+),CD45(−),CD309(+)	= EPC number
Ligi et al. (Ref. 93)	CB	EPCs ECFCs	CD34(+),CD45(−) CD34(+),CD45(−),CD31(+),CD144(+), CD146(+), CD309(+), CD14(−), CD41(−)	= EPC number ↓ ECFC colony number
Paviotti et al. (Ref. 94)	PB/CB	EPCs	CD 34(+),CD309(+); CD133(+),CD309(+); CD34(+), CD133(+), CD309(+)	= EPC number
*Intrauterine growth restriction*				
Sipos et al. (Ref. 100)	CB	ECFCs CPCs	7AAD(−),CD31(+),CD45(−),CD309(+),CD34(+) 7-AAD(−),CD31(−),CD45(+),CD133(+),CD34(+)	↓ ECFC number, Proliferation ↓ CPC number
Monga et al. (Ref. 83)		EPCs	CD45(Low),CD34(+),CD133(+) CD45(d),CD133(+)CD34(−)	↓ EPC number = EPC number
Hwang et al. (Ref. 103)	CB	EPCs	CD133(+),CD309(+),CD34(+)	↓ EPC number

CB: cord blood; CPCs: circulating progenitor cells; CECs: circulating endothelial cells; CACs: circulating angiogenic cells: d: diminished; EPCs: endothelial progenitor cells; ECFCs: endothelial colony-forming unit cells; n.A.: not assessed; PB: peripheral blood; ‘= EPC number’: no significant differences in EPC numbers.

#### Preeclampsia

The severe hypertensive pregnancy disorder, preeclampsia, is associated with endothelial dysfunction (Ref. 49) and is believed to contribute to early vascular ageing processes (Ref. 50). This condition can impact both the mother and the offspring later in life, leading to CV morbidity (Ref. 51).

The odds for chronic hypertension, CADs and death are doubled in mothers with a preeclamptic history (Ref. 52). It is assumed that with the release of free radicals and vasculotoxic factors of the placenta into the maternal circulation, chronic inflammation is triggered, contributing to endothelial damage and dysfunction (Ref. 53). In various publications, a reduced number and function of EPCs from preeclamptic women have been described. A lower amount of CFU-ECs was detected in women with preeclampsia compared to gestational-age-matched healthy controls (Refs 32, 37, 54, 55). Luppi *et al*. described a lower number of CFU-ECs and CACs (Ref. 56). In contrast, the number of EPCs, specifically the CACs subpopulation defined as CD34(+)CD133(+)CD309(+), in peripheral blood from 20 non-pregnant women, 36 women with normal pregnancies and 10 women with preeclampsia did not differ significantly between the preeclamptic and normal pregnancy groups. Interestingly, Angiotensin II-induced proliferative activity of CACs was significantly increased in the preeclamptic group (140.0 ± 17.2%) compared with that in the third trimester of normal pregnancy (87.4 ± 4.4%) (p < 0.05) (Ref. 35).

Few studies have specifically investigated the number of ECFCs in the peripheral blood of preeclamptic women. In a prospective study comparing the numbers of all EPC subsets across the three trimesters of normal and preeclamptic pregnancies, the number of CFU-ECs was found to be reduced (p = 0.039), while CACs and ECFCs were higher in women with preeclampsia (p = 0.014). Statistical significance was achieved only by ECFCs characterized by a CD309(+)CD45(−) profile, while the CD34(+)CD45(−) and CD34(+)CD133(−)CD309(+) defined ECFC populations did not reach statistical significance (Ref. 37) (Table 1).

Most of the studies mentioned here report conflicting results regarding EPC counts, which can be attributed to variations in study design and the different methods used to identify EPC subtypes, including CFU-ECs, CACs and ECFCs. Consequently, the use of EPCs as a biomarker for assessing endothelial impairment in preeclampsia is still in its early stages and requires further large-scale prospective studies, along with clearly defined markers for EPCs and EPC subtype classification.

#### Gestational diabetes mellitus

Gestational diabetes mellitus (GDM) is a type of diabetes that develops during pregnancy, resulting in elevated blood glucose levels. If left untreated, GDM can lead to health complications for both mother and infant. Women with GDM are at two-fold increased risk of CVD, including hypertension and CAD (Ref. 57). By evaluating circulating EPCs in pregnant women with gestational alterations of glucose tolerance, Penno *et al.* found a decrease of maternal EPC counts (defined as (CD34(+)CD309(+) cells) (30.2 ± 22.3 versus 60.8 ± 59.6) (p = 0.010)) and CD34(+)CD133(+)CD309(+) (CACs) cells (26.5 ± 20.4 versus 55.6 ± 57.8 (p = 0.011)) in GDM compared to normal pregnant controls (Ref. 58). This was in line with a study demonstrating lower numbers of maternal CACs (defined as CD34(+)CD133(+)CD309(+) cells) in women with GDM as compared to normoglycemic controls (0.26% versus 0.41% (p < 0.05)) (Ref. 59). Further, increased soluble adhesion molecules, decreased superoxide dismutase (SOD) expression and increased endothelial nitric oxide synthase (eNOS) expression was observed in women with GDM (Ref. 59). Although decreased SOD expression and increased eNOS expression was also detectable in foetal GDM-derived CACs the number of circulating foetal CACs in UCB showed no significant changes (1.76% versus 1.46%) (Ref. 59).

Exposure of the foetus to GDM predisposes children to future health complications such as high blood pressure and CVD (Refs 60–62). This is thought to be due to a functional impairment of EPCs, including ECFCs (Ref. 63). Ingram and colleagues showed that *in vitro* hyperglycaemia and a diabetic intrauterine environment reduced ECFC colony formation, self-renewal capacity and capillary-like tube formation, which was linked to premature senescence and reduced proliferation of the cells (Ref. 64). This finding was further supported by Gui *et al*., who demonstrated that vitamin D_3_ treatment rescued ECFC dysfunction in pregnancies affected by GDM (Ref. 65). In addition, foetal ECFCs exposed to GDM have been shown to exhibit decreased vasculogenic potential and altered gene expression, including a significant increase in transgelin, which contributes to vascular dysfunction (Ref. 63). In contrast to these studies, a decrease of circulating progenitor cells (CPCs), defined as CD34(+)CD133(+)CD45(+)CD31(+), was detected in maternal blood and UCB from GDM pregnancies compared to healthy controls. However, no difference was found in the numbers of CACs and ECFCs in UCB from GDM pregnancies (Ref. 66) (Table 1).

Based on these results, it can be concluded that GDM affects maternal EPCs. However, there is a need for further studies to investigate its potential impact on the foetus.

### EPCs, neonatal morbidity and future cardiovascular risk – insights from the foetal side

#### Preeclampsia

Numerous studies indicate that an adverse perinatal environment along with pregnancy complications and related conditions contributes to an increased risk of CVD in adulthood (Ref. 50). Current literature suggests growing evidence of long-term CV sequelae in children exposed to preeclampsia *in utero* (Ref. 67). Offspring with a maternal history of preeclampsia are at higher risk of developing hypertension and elevated BMI (Ref. 68) during childhood and adolescence. Additionally, these individuals face an increased risk of stroke (Refs 68–70), elevated systolic and diastolic blood pressure (Refs 71–73) and greater stiffness in the peripheral vascular system (Ref. 74). Notably, the pathological and endothelial-damaging factors associated with hypertension have been demonstrated in the maternal circulation during preeclampsia (Ref. 75), but have not yet been identified in the foetal blood vessels. This suggests that additional mechanisms may be responsible for the development of persistent foetal endothelial dysfunction. Some studies indicated that similar changes occur in the UCB of offspring exposed to preeclampsia, mirroring those observed in maternal circulation. These changes include elevated levels of anti-angiogenic and pro-inflammatory molecules such as soluble fms-like tyrosine kinase-1 (sFlt1), soluble endoglin (sENG), IL-6 and tumour necrosis factor (TNF)-alpha (Refs 76–79).

The function and survival of ECFCs may be influenced by this altered angiogenic and inflammatory environment. Additional evidence suggests that the migration, angiogenic and proliferation capacity of ECFCs from preeclamptic offspring (Refs 80–82) are impaired, potentially contributing to future CVD. In an observational study by Muñoz-Hernandez *et al.*, a decrease in the number of ECFC colonies in UCB from preeclamptic pregnancies was noted compared to normotensive pregnancies; however, cellular function remained intact when these cells were transfected into immunodeficient mice (Ref. 83). Conversely, while the number of ECFC colonies in UCB tended to be lower in preeclampsia, the difference was not statistically significant (Ref. 84). Likewise, Monga *et al*. did not find a significant association between preeclampsia and EPC counts (Ref. 85) (Table 1). In another study, comparisons were made between capillary-tube formation, proliferation and migration capacity of foetal ECFC from preeclamptic women and those of gestational age-matched healthy controls in a functional *in vitro* analysis. (Ref. 82). Additionally, when exposed to serum from preeclamptic UCB ECFCs demonstrated reduced ability to integrate and invade into the capillary-like networks of mature endothelial cells (Ref. 86), highlighting the impact of preeclampsia on ECFC functionality. Interestingly, this effect was reversed by vitamin D_3_ treatment.

The discrepancies in methodologies and the characterization of molecular markers for ECFCs and EPCs by flow cytometry likely account for these varying results. However, the extent to which the limited or reduced function of EPCs, particularly ECFCs, affects the CV health of preeclamptic offspring has not yet been fully established and requires further investigation.

#### Preterm birth and bronchopulmonary dysplasia

Defined as birth before the 37th week of gestation, preterm birth can lead to various complications, with bronchopulmonary dysplasia (BPD) being one of the most significant. BPD is a chronic lung disease that primarily affects very premature infants, particularly those with low birth weight. It impacts up to 45% of babies born before 29 weeks of gestation (Ref. 87). Additionally, preterm birth is associated with an increased risk of high blood pressure in adulthood (Ref. 88). A correlation has been observed between dysfunction of ECFCs in preterm infants and the development of BPD (Refs 84, 89).

The number of ECFCs in UCB was significantly reduced in preterm infants who went on to develop moderate or severe BPD, compared to those who did not (p < 0.001) (Ref. 84). In a cross-sectional observational study, ECFCs isolated from 55 preterm-born young adults were compared with full-term controls. In the preterm-born subjects, elevated systolic blood pressure significantly correlated with reduced ECFC proliferation (p = 0.03) and number of branching points in a tube formation assay (p = 0.039) (Ref. 90). This impaired ECFC function was also associated with BPD (Ref. 90). Further research has explored the relationship between EPC count, function and preterm birth, yielding contradictory results. Two studies found that EPC counts were increased in the peripheral blood of individuals born preterm compared to those born full-term at various intervals after birth (Refs 91, 92).

The observational study by Safranow and colleagues provides valuable insights into the differences in the counts of early and late EPCs (defined as mature EPCs with the marker expression of CD133(−)CD34(+)CD144(+)) between 90 preterm and 52 full-term infants at birth, 2 and 6 weeks after birth. The study found that preterm infants had higher counts of both early- and late-outgrowth EPCs in their UCB compared to full-term infants (Ref. 92). This suggests that preterm infants may have a different endothelial cell development profile at birth. Likewise, another study examined peripheral blood samples from preterm infants collected at 10 weeks, comparing those with retinopathy of prematurity (N = 29), preterm infants without retinopathy of prematurity (N = 29) and healthy full-term infants (N = 30). The results indicated that preterm infants with retinopathy of prematurity had significantly higher counts of early EPCs (CD34(+)CD133(+)CD144(+)) in their peripheral blood compared to those without retinopathy of prematurity (Ref. 91). This finding may suggest a potential link between EPC levels and the development of retinopathy of prematurity in preterm infants. However, it is important to note that other studies have reported no differences in EPC or ECFC counts (Refs 85, 93–95) between preterm and full-term infants. In contrast, preterm ECFCs from UCB were found in greater numbers and to proliferate more rapidly but were more susceptible to hyperoxia compared to term ECFCs (Ref. 93).

The research conducted by Ligi *et al.* focused on the flow cytometric measurement of EPCs in UCB, specifically analysing the CD34(+) and CD45(−) markers. Their findings indicated no significant difference in the number of these cells between preterm and term births. However, they did observe a reduction in the colony-forming ability, proliferation and angiogenic capacity of ECFCs (characterized by the markers CD34(+)CD45(−)CD14(−)CD41(−)CD31(+)CD146(+)CD144(+)CD309(+)) in both *in vitro* and *in vivo* settings (Ref. 95). Additionally, Pavigotti and colleagues found no detectable link between the number of EPCs and the incidence of BPD (Ref. 96) (see Table 1). These discrepancies in findings may be attributed to the variability in the characterization of ECFCs or EPCs across studies, particularly regarding the different cell surface markers used for measurement. This variability could help explain the inconsistencies observed in the analyses. Overall, EPCs were found to be either comparable to or increased in preterm infants. There is compelling evidence suggesting an association between reduced numbers of EPCs or ECFCs and the development of BDP, which may position EPCs as potential prognostic marker for this complication associated with preterm birth.

#### Intrauterine growth restriction

Clinically, intrauterine growth restriction^2^ (IUGR) is defined as the pathologically reduced growth of a foetus during pregnancy, characterized by a foetal estimated weight falling below the 10th percentile throughout pregnancy (Refs 97, 98). IUGR is observed in approximately 10–15% of pregnancies (Refs 97, 99). The implications of IUGR are complex, potentially resulting in both short-term and long-term health consequences for the child.

Infants born with IUGR face an elevated risk of prematurity, low birth weight, respiratory distress syndrome, hypoglycaemia and challenges in temperature regulation (Refs 97, 100). In addition, IUGR is linked to an increased risk of CVD later in life (Ref. 101). Children born with IUGR are more likely to experience alterations in vascular structure and function, which can contribute to high blood pressure, CAD and other CV issues. In arterial cord blood derived from pregnancies complicated by IUGR, the number of ECFCs was significantly lower (p = 0.02), as was the count of CPCs (p = 0.044) compared to normal pregnancies. Further, IUGR-ECFCs displayed reduced proliferation (p = 0.01), migration (p = 0.007) and diminished chemotactic abilities to stromal cell-derived factor 1 (p = 0.007) coupled with reduced hypoxia-induced matrix metalloproteinase-2 release (p = 0.02) (Ref. 102). Approximately 22% of pregnancies affected by preeclampsia are associated with IUGR (Ref. 103). In a large case control study, the rates of IUGR <10% (defined as foetal growth using gestational age at delivery and actual birth weight) in patients with mild preeclampsia (N = 61), severe preeclampsia (N = 117) and controls (N = 125) were 28%, 34% and 12% (Ref. 104).

Early-onset preeclampsia can impair placental growth and lead to insufficient nutrient supply to the foetus, resulting in IUGR. In an observational study comparing EPCs from 19 preterm and 27 term deliveries CD45(d)CD133(+)CD34(−) cell numbers were significantly increased ([0.43, (0.06–1.38)], [median, (range)]) (p = 0.002) in presence of preeclampsia. Conversely, CD45(d)CD133(+)CD34(+) cell numbers were lower in cases of IUGR ([0.2 (0–2.46)] [median, (range)]) (p = 0.031) while no changes were observed in CD45(d)CD133(+)CD34(−) cells (Table 1). Notably, differences in EPC types associated with preeclampsia and IUGR were only evident in term UCB (Ref. 85). Hwang and colleagues demonstrated that foetal EPC counts in pregnancies complicated by IUGR (N = 30) were significantly lower, and the differentiation time of EPCs was prolonged. Likewise, the staining intensity of senescence-associated beta galactosidase (SA-β-gal) was relatively increased, while telomerase activity in EPCs was significantly decreased (70.3 ± 6.5% versus 100.0 ± 9.4%) (p < 0.001)) compared to normal pregnancy (Ref. 105).

Although IUGR and low birth weight (LBW) are clinically distinct concepts, they are often interrelated. Ligi *et al*. investigated ECFC function in 25 preterm neonates with LBW compared to term controls. Their findings revealed that LBW was associated with a reduced number of colonies formed by ECFCs and a delayed appearance of their clonal progeny. Moreover, LBW-ECFCs exhibited diminished capacity for sprout and tube formation, as well as reduced migration and proliferation *in vitro.* This angiogenic deficiency was further confirmed *in vivo*, as LBW-ECFCs were unable to form robust capillary networks in Matrigel plugs injected in mice (Ref. 95).

In summary, foetal EPCs in cases of IUGR and reduced foetal growth are diminished in number, exhibit lower differentiation capacity, and have an increased proportion of senescent cells. These factors may help explain the long-term CV outcomes for children born from IUGR pregnancies; however, further mechanistic studies are needed to validate these findings.

### Premature ageing of EPCs in pregnancy-related diseases and pathologies

To date, there has been limited research on the role that premature ageing of EPCs or specifically ECFCs may play in the development of diseases throughout life. Early cell ageing is a highly complex molecular process characterized by various factors, including cellular senescence. Senescent cells and the process associated with premature cellular ageing can negatively affect the microenvironment through the secretion of soluble factors and the release of extracellular vesicles, both of which play a significant role in CVD (Ref. 106). As individuals age, the numbers and functionality of EPCs and ECFCs decline due to cellular senescence and chronic inflammation. This decline contributes to an increased risk of age-related vascular diseases, such as atherosclerosis and impaired wound healing. Research aimed at rejuvenating these cells, particularly in elderly and diseased populations, holds promise for future regenerative therapies targeting CV and vascular conditions (Refs 8, 107).

An adverse pregnancy environment and early programming of EPCs may explain the link between premature cell ageing and its effects on endothelial health, as well as the associated increased CV risk. Research by Vassallo *et al*. demonstrated that sirtuin-1 (SIRT-1) deficiency accelerates senescence and impairs ECFC function in neonates born preterm (Ref. 108). Moreover, preterm ECFCs exhibited a senescence-associated secretory phenotype (SASP) linked to the biogenesis of pro-senescent microparticles, driven by a SIRT1-dependent epigenetic regulation of mitogen-activated protein kinase kinase 6 (MKK-6), which may promote senescence in early-passage umbilical vein endothelial cells. (Ref. 106). In a rat model of foetal growth restriction (FGR), ECFCs isolated from the bone marrow of FGR males showed reduced numbers, impaired proliferation and diminished ability to form capillary-like structures, all associated with oxidative stress and stress-induced premature senescence (Refs 9, 109). In addition, a significant link has been observed between telomere length, a key factor in biological ageing and the replating capacity of ECFCs in relation to atherogenesis. Toupance and colleagues found a positive correlation between telomere length and the replating capacity of ECFCs. Individuals with longer telomers exhibited a higher number of self-renewing ECFCs suggesting that they may possess a better capacity for endothelial repair (Ref. 110). While the association between telomere length and pregnancy complications, such as preeclampsia and GDM, has been explored in some studies (Refs 111, 112), these investigations primarily focused on leucocyte telomere length and did not examine ECFCs or other EPC subtypes. Therefore, further studies investigating the relationship between telomere length in ECFCs and pregnancy complications would be particularly valuable.

## Epigenetic regulation

Although EPCs, particularly ECFCs, hold promise as biomarkers for certain pathological changes and for facilitating the revascularization of damaged tissue, their clinical application remains unestablished due to the complexity of the various subtypes. Before EPCs are used as a cell therapy in humans, it is essential to identify and elucidate the underlying mechanisms and multiple signalling networks involved. Over the past few years, significant progress has been made in understanding the epigenetic regulation of EPCs in several pregnancy-associated disorders. Insights into this epigenetic programming can pave the way for the development of new diagnostic approaches and treatment strategies, particularly concerning the long-term consequences for both mother and offspring. Epigenetic regulation is of particular interest as it may influence the angiogenic function of EPCs. In the following chapter, we will discuss the changes in epigenetic profiles and the associated transcriptomic alterations of maternal and offspring EPCs in relation to various pathological changes during pregnancy.

### Epigenetic regulation of EPCs in pregnancy-related diseases and pathologies

An adverse perinatal environment can significantly influence future health outcomes. The epigenome, which is highly responsive to the molecular environment, may play a crucial role in this process. It is believed that an altered molecular environment leads to changes in the epigenetic programming of EPCs during early pregnancy development, potentially paving the way for subsequent diseases such as hypertension and other CV risk factors. A detailed proteomic comparison was conducted between functional and dysfunctional ECFCs, defined as CD45(−)CD31(+)VEGFR-2(+)CD34(+), from 11 subjects (45% males, age 27 ± 5 years). Hierarchical functional cluster analysis revealed distinct proteomic signatures between functional and dysfunctional ECFCs, highlighting changes in cellular mechanisms related to exocytosis and vesicle trafficking, extracellular matrix organization, cell metabolism and apoptosis. Notably, secreted protein acidic and rich in cysteine (SPARC), CD36, lumican (LUM) and pentraxin 3 (PTX3) were identified as anti-angiogenic proteins in dysfunctional ECFCs, which were associated with impaired angiogenic capacity and expansion (Ref. 113). The epigenetic regulation of EPCs in pregnancy-related diseases and pathologies will be discussed below.

#### Preeclampsia

Reduced angiogenesis capacity of EPCs has been documented in various studies, particularly in the context of preeclampsia. The genomic methylation patterns of foetal ECFCs from preeclamptic pregnancies, which exhibit altered angiogenesis, showed changes at 1,266 CpG sites at passage 3 and at 2,362 sites at passage 5 compared to ECFCs from normal pregnancies. Differential methylation of genes can impact cell metabolism, cell cycle and transcription, leading the authors to hypothesize that epigenetically altered EPCs may have significant implications for both normal morphogenesis and postnatal vascular repair capacity (Ref. 114). In support of this, a comparative microRNA profiling study of ECFCs from UCB (N = 6) and maternal blood (N = 6) of preeclamptic pregnancies, as well as ECFCs from UCB and maternal blood (both N = 6) from healthy women, revealed differentially expressed miRNAs across all groups. Notably, hsa-miR-1270 levels were significantly different. Inhibiting hsa-miR-1270 in foetal ECFCs reduced their tube-forming ability and chemotactic motility, although it did not affect proliferation (Ref. 115). Additionally, the downregulation of hsa-miR-1270 levels was associated with increased levels of ataxia telangiectasia mutated (ATM), a key kinase in DNA double-strand repair (Ref. 116). Satoh *et al*. observed that the shortening of EPC telomeres due to increased oxidative DNA damage could play a critical role in the development of CAD (Ref. 117). Another study investigated oxidative stress and DNA damage in mild preeclampsia and its effects on offspring, finding increased DNA damage in both the mothers and their children (Ref. 118). However, it is important to note that this study focused on mononuclear leukocytes from UCB and maternal blood, rather than on EPCs.

#### Gestational diabetes mellitus

Epigenetic regulation has been increasingly linked to impaired foetal ECFCs in cases of GDM. A genome-wide mRNA expression analysis conducted on ECFCs from control and GDM pregnancies revealed numerous genes with abnormal expression patterns in GDM-derived ECFCs (Ref. 119). Specifically, 38 genes were found to be differentially expressed between control and GDM exposed ECFCs. Notably, placenta associated 8 (PLAC8) protein was highly expressed in ECFCs from GDM pregnancies, and its expression correlated with maternal hyperglycaemia. The methylation status of 17 CpG sites in the *PLAC8* gene negatively correlated with mRNA expression. Furthermore, knockdown of *PLAC8* in GDM-exposed ECFCs improved defects in proliferation and senescence (Ref. 119). Likewise, based on this genome-wide screen, elevated levels of transgelin (TAGLN) mRNA were identified in foetal ECFCs from pregnancies complicated by GDM, which impaired ECFCs migration, cell alignment and network formation (Ref. 63).

#### Preterm birth

Similar observations have been made regarding the induction of a distinct transcriptome profile in ECFCs due to preterm birth. Hierarchical clustering identified 253 genes that were upregulated and 476 genes that were downregulated in preterm ECFCs (≥1.5 fold-change) (p ≤ 0.05) compared to term controls. Microarray analysis indicated that SIRT1 deficiency regulates the MKK6/p38 mitogen-activated protein kinase (MAPK)/Heat shock protein 27 (Hsp27) pathway, which enhances endothelial microparticle biogenesis in senescent ECFCs (Ref. 106). Vinci and colleagues demonstrated that altered angiogenic function in ECFCs is associated with decreased expression of pro-angiogenic genes, with *angiomotin* (*AMOT*) identified as a strong positive regulator of angiogenesis. A comparative analysis of the DNA methylation profile of the promoter CpG island of the *AMOT* gene in foetal ECFCs from 16 preterm newborns (28–35 weeks gestational age) and 15 term newborns revealed that the methylation rate of the *AMOT* gene was significantly higher in preterm infants (4.5% versus 2.5%). This suggests that epigenetic mechanisms may play a crucial role in regulating angiogenesis during development (Ref. 120).

In summary, adverse environmental conditions during gestation may induce epigenetic changes in EPCs. Therefore, exploring these molecular mechanisms is crucial, and further studies are needed to elucidate how dysfunctional EPCs are linked to genetic regulation that may contribute to the development of future diseases.

## Summary

EPCs are essential for vascular repair and regeneration, significantly contributing to angiogenesis and vasculogenesis. Since their discovery in the late 1990s, various EPC subtypes have been identified, with ECFCs playing a crucial role in postnatal neovascularization. EPCs are derived from multiple sources, including bone marrow, peripheral blood and UCB, with their functionality influenced by factors such as gestational age, hormonal changes and environmental conditions. During pregnancy, EPC levels fluctuate, with oestrogen promoting their mobilization and delaying cellular senescence. However, EPCs’ function declines with age, leading to impaired vascular repair and increased CV risks.

EPCs have been implicated in numerous diseases, including ischaemic conditions, pulmonary arterial hypertension and cancer. In pregnancy-related disorders such as preeclampsia and GDM, EPC dysfunction is linked to endothelial impairment and long-term CV risks for both mothers and offspring. Reduced EPC numbers and altered functionality are associated with conditions such as preterm birth, IUGR and BPD, highlighting their importance in foetal development. Additionally, epigenetic regulation plays a significant role in EPCs’ behaviour, with adverse pregnancy environments inducing molecular changes that may predispose individuals to future CVD.

## Conclusion and future directions

EPCs are crucial for vascular health with their dysfunction linked to various pregnancy-related complications and long-term CV risks. While their potential as biomarkers and therapeutic targets is promising, further research is needed to fully understand their role in endothelial repair and disease progression. Investigating epigenetic mechanisms and molecular pathways in EPC regulation could lead to novel therapeutic strategies for pregnancy-related disorders and CV conditions. Expanding clinical studies will be essential to translating these findings into effective diagnostic and treatment approaches, ultimately improving maternal and neonatal health outcomes.

Future research on EPCs should focus on clarifying the molecular mechanisms underlying EPC’s role in vascular repair, particularly in pregnancy-related disorders. Given the conflicting findings on EPC dynamics in conditions such as preeclampsia, GDM and IUGR, larger and more standardized clinical studies are needed to confirm their potential as biomarkers. Investigating the impact of epigenetic modifications on EPC functionality may provide insights into how adverse pregnancy environments contribute to long-term CV risks in both mothers and offspring. Further exploration of therapeutic interventions, such as pharmacological agents or lifestyle modifications, to enhance EPC mobilization and function could pave the way for novel treatment strategies targeting endothelial dysfunction. Overcoming challenges related to cell heterogeneity, standardization of isolation techniques and optimizing their regenerative potential will be key to advancing EPC-based therapies and improving maternal and neonatal health outcomes.

## Footnotes

1 While the mobilization of EPCs from the bone marrow and their circulation in peripheral blood is well documented in mice, direct evidence of their in vivo differentiation into mature endothelial cells in humans remains limited. Most functional insights are derived from in vitro studies, where EPCs exhibit endothelial characteristics under culture conditions. Although animal models have demonstrated true endothelial differentiation, confirming these findings in humans poses ethically and technically challenges, and the current evidence for human bone marrow-derived and circulating EPCs remains largely indirect.

2 The terms IUGR and FGR refer to the same pathologic delay of fetal growth in utero. IUGR was more commonly used in former medical literature, whereas FGR is now more prevalent in current guidelines and academic literature. In our manuscript we have used the original terms cited by the authors.
